# Supplementary material for: Broadband miniaturized spectrometers with a van der Waals tunnel diode
Source: Nat Commun. 2024 Jan 17;15:571. doi: 10.1038/s41467-024-44702-8 (PMC10794200; doi:10.1038/s41467-024-44702-8)
Supplement: Supplementary file 1 — Supplementary information [file 41467_2024_44702_MOESM1_ESM.pdf]

Supplementary Information

Broadband miniaturized spectrometers with a van der Waals tunnel diode

*MD Gius Uddin[1,2], Susobhan Das[1], Abde Mayeen Shafi[1], Lei Wang[3], Xiaoqi Cui[1,2], Fedor Nigmatulin[1,2], Faisal Ahmed[1], Andreas C. Liapis[2], Weiwei Cai[3], Zongyin Yang[4], Harri Lipsanen[1], Tawfique Hasan[5], Hoon Hahn Yoon[1,6], Zhipei Sun[1,2]**

[1]Department of Electronics and Nanoengineering, Aalto University, Espoo 02150, Finland.
[2]QTF Centre of Excellence, Aalto University, Aalto 00076, Finland.
[3]Key Lab of Education Ministry for Power Machinery and Engineering, School of Mechanical Engineering, Shanghai Jiao Tong University, Shanghai 200240, China.
[4]College of Information Science and Electronic Engineering and State Key Laboratory of Modern Optical Instrumentation, Zhejiang University, Hangzhou 310027, China.
[5]Cambridge Graphene Centre, University of Cambridge, Cambridge CB3 0FA, UK.
[6]Department of Semiconductor Engineering, School of Electrical Engineering and Computer Science, Gwangju Institute of Science and Technology, 123 Cheomdangwagi-ro, Buk-gu, Gwangju 61005, Republic of Korea.
*Corresponding author: zhipei.sun@aalto.fi

S1. Structural characterization of BP/MoS_2 heterostructure.

The tapping-mode AFM topography of the heterostructure is provided below. Thicknesses of the BP and the MoS_2 flakes are measured as ~45 nm and ~33 nm, respectively. The inset shows the corresponding AFM map with a blue dashed line indicating the path of the AFM line scan.

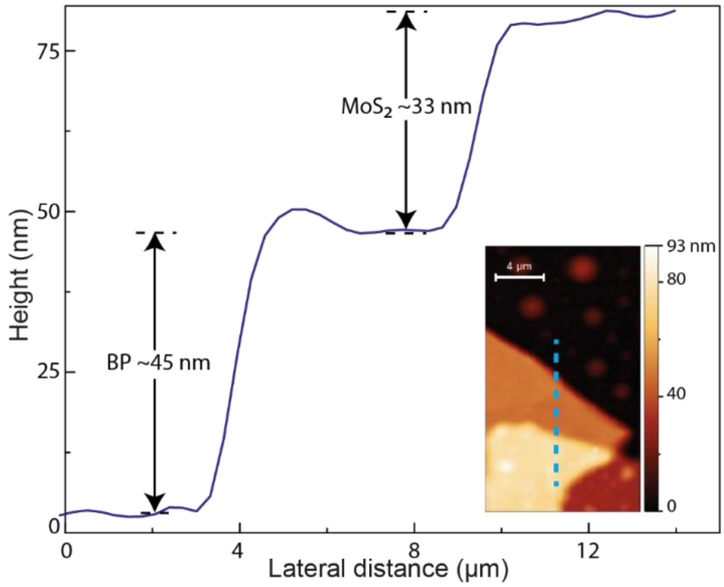

Figure S1: AFM topography of BP/MoS_2 heterostructure.

S2. Optoelectronic characterization of BP and MoS_2.

Figure S2 shows transfer curves of BP (red line) and MoS_2 (black line) flakes. MoS_2 flake exhibits typical n-type properties with an on-off ratio of ~10^6. Very weak gate dependence of BP indicates its degenerate p-type behavior.

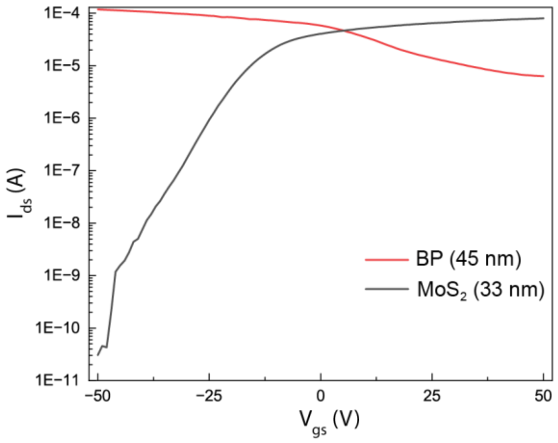

Figure S2: Transfer characteristics of BP and MoS_2.

The MoS_2/BP van der Waals tunnel diode is highly sensitive to optical excitation and the noise-equivalent-power (NEP) of the device is on the order of ~nanowatt per square root of hertz, which is comparable to that of the previously reported BP photodetectors[1].

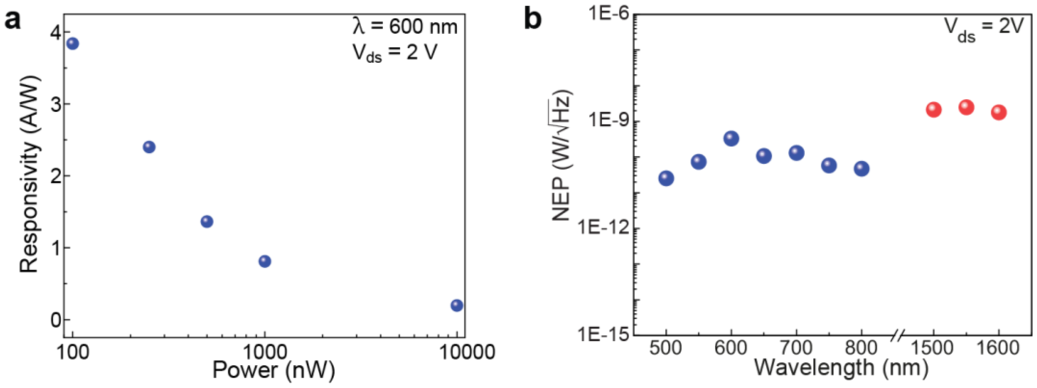

Figure S3: (a) Responsivity of a MoS_2/BP tunnel diode under different optical powers, and (b) NEP under visible and NIR light illumination.

S3. The spectrum reconstruction process

We reconstruct the unknown spectrum S(λ) from the measurement dataset. The desired spectrum functions relate to the generated currents in the device. This can be expressed as:

$$\int_{\lambda_{\min}}^{\lambda_{\max}} S(\lambda) R_i(\lambda)\, d\lambda = I_i \tag{S1}$$

where $i = 1, 2, \ldots, n$ and denotes the index of the measurement (in our case each measurement corresponds to the certain value of the bias voltage V_{ds}). In the Eq. S1, I_i refers to the measured photocurrent, $R_i(\lambda)$ is the response function, λ_{min} and λ_{max} are minimum and maximum wavelength, respectively.

To extract the function S(λ), we use a linear combination of Gaussian basis functions ($\varphi_j(\lambda)$) with wavelength-independent weight coefficients α_j:

$$S(\lambda) \approx \sum_{j=1}^{m} \alpha_j\, \phi_j(\lambda) \tag{S2}$$

It is natural to choose Gaussian functions as a basis set for the expansion:

$$\phi_j(\lambda) = \frac{1}{\sigma\sqrt{2\pi}}\, exp\left[-\frac{1}{2}\left(\frac{\lambda - \lambda_j}{\sigma}\right)^2\right] \tag{S3}$$

where σ is the full width at half maximum (FWHM) of the Gaussian function and λ_j denotes the peaks of the Gaussian curves. The Gaussian peaks are generated as a linearly spaced vector within the range of the minimum and maximum wavelength:

Discretization of the problem allows us to represent Eq. (S1) in a simple matrix form of the system of algebraic equations:

$$\widehat{R} \cdot \widehat{\varphi} \cdot \vec{\alpha} = \vec{I}_{ph} \tag{S4}$$

To solve the obtained matrix equation, one needs to minimize a residual norm (squared error) by solving non-negative least square problem:

$$\min_{\vec{\alpha}} \left\| \widehat{R} \cdot \widehat{\varphi} \cdot \vec{\alpha} - \vec{I}_{ph} \right\|_2^2 \tag{S5}$$

We use Tikhonov regularization to avoid instabilities in the solution due to the high-frequency noise signals. In this method, one needs to add an auxiliary term to the residual norm. This term is defined by the regularization factor whose optimal value can be found with Generalized Cross Validation adaptive method. A set of coefficients is determined by the condition of the global minimum of the modified residual norm. Finally, the substitution of the found weight coefficients in Eq. S2 allows to reconstruct the spectrum of the unknown incident light.

S4. Broadband reconstruction

We assess the performance of our spectrometer in broadband spectra reconstruction. The black and red solid lines in Fig. S4 represent the spectra reconstructed with our spectrometer and measured using a commercial spectrometer (FLAM-S, Ocean Optics), respectively. The results show that our spectrometer agrees with a commercial spectrometer regarding wide spectral measurement.

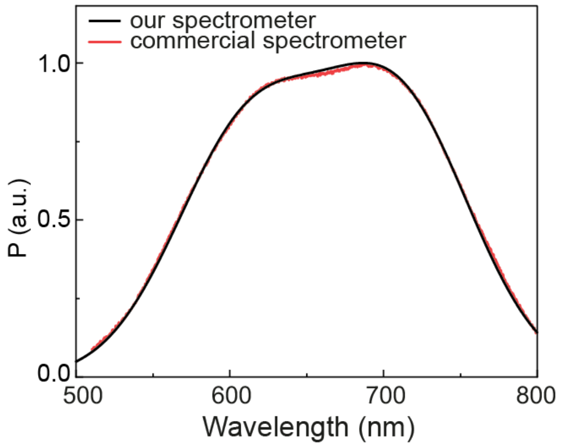

Figure S4: Broadband spectrum measurement comparison between our spectrometer and a commercial spectrometer. The full-width half maximum of the spectrum is ~180 nm.

S5. Spectrometer performance at the NIR range with larger learning step.

To demonstrate the performance of our spectrometer at the NIR range, we first obtain the responsivity matrix experimentally in the learning process for monochromatic incident lights of wavelength ranging from 1500 to 1600 nm in the step of 10 nm. Our broadband spectrometer effectively resolves the NIR signals, as shown in the figure below. Accuracy compromise due to larger learning steps agrees well with the previous report[2].

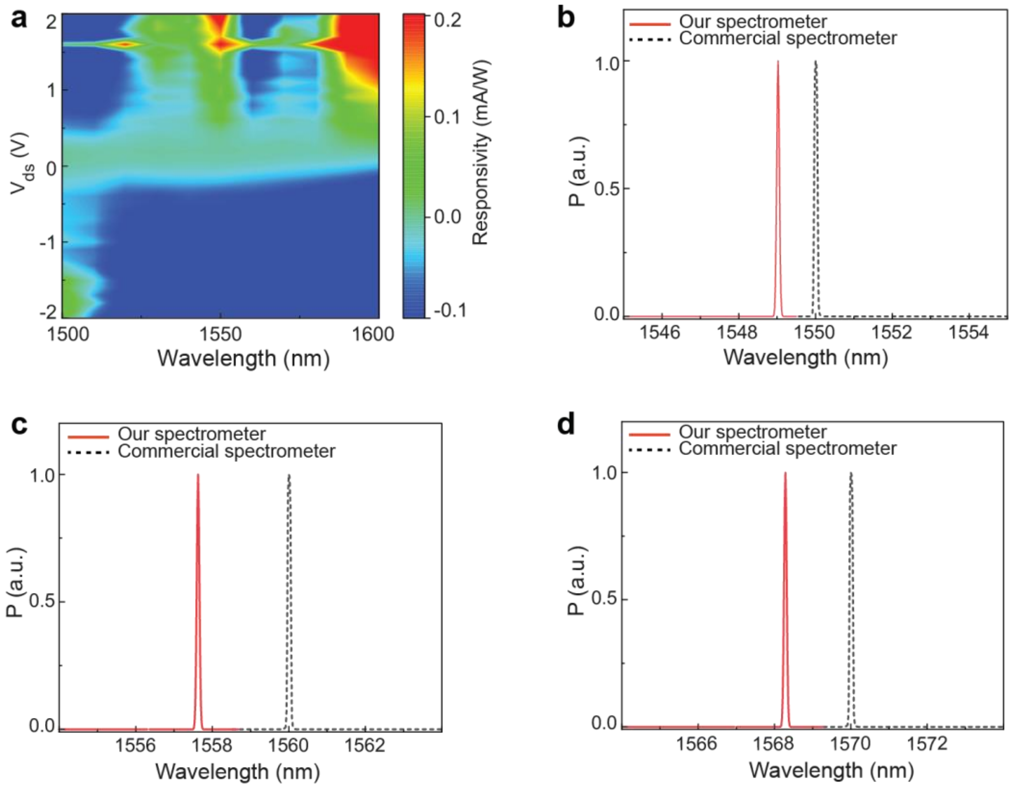

Figure S5: Spectrometer at the NIR range for larger steps during the learning process. (a) Color contour plot of spectral response matrix. (b-d) Reconstruction of quasi-monochromatic spectra with our spectrometer (solid curves). Dashed curves represent corresponding spectra measured with a commercial spectrometer.

S6. Operational stability and reproducibility of the spectrometer

Figure S6 shows the operational stability and reproducibility of our spectrometer. Figure S6(a) compares the current density of a freshly prepared BP/MoS_2 tunnel diode with that of the same device after one month under dark conditions. The excellent stability of the device refers to high-quality passivation of the BP/MoS_2 heterostructure. Further, our spectrometers show operational stability under repeated measurements of identical light sources (Fig. S6(b)). The results show that the device is stable within ~10 days. Figure S6(c-d) benchmark the performance of our best two spectrometers against the commercial spectrometers. The device-to-device variation is reasonable, such as ~1.2 nm in the visible range (Fig. S6(c)), and ~0.02 nm in the NIR range (Fig. S6(d)).

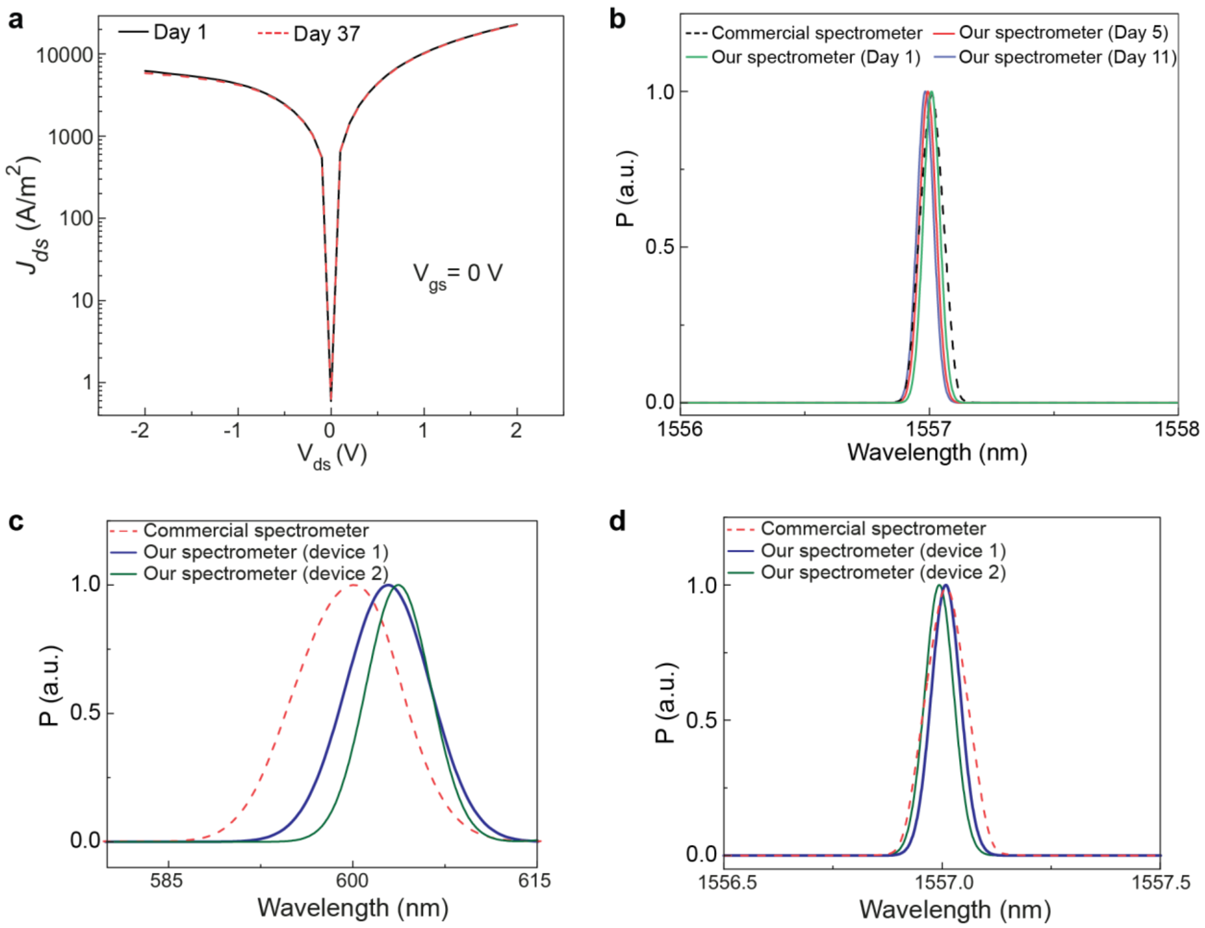

Figure S6: Operational stability and device-to-device variation of our spectrometer. (a) Comparison of dark current density over a month. (b) Operational stability of the spectrometer under repeated measurements of identical light sources. (c-d) Comparison of spectral reconstruction performance of our best two devices working in the visible and the NIR ranges.

References

[1] L. Huang et al., Infrared Black Phosphorus Phototransistor with Tunable Responsivity and Low Noise Equivalent Power. ACS Appl. Mater. Interfaces, 9, 36130 (2017).

[2] H. H. Yoon, et al., Miniaturized spectrometers with a tunable van der Waals junction. Science 378, 296-299 (2022).
